# Long non-coding RNA AK058003, as a precursor of miR-15a, interacts with HuR to inhibit the expression of Υ-synuclein in hepatocellular carcinoma cells

**DOI:** 10.18632/oncotarget.14276

**Published:** 2016-12-27

**Authors:** Xiaoqin He, Yongfa Zheng, Yuefeng Zhang, Yuanyuan Gan, Yujie Zhou, Huilin Liang, Dongcheng Wu, Wei Ge, Junjian Deng, Ximing Xu

**Affiliations:** ^1^ Cancer Center, Renmin Hospital of Wuhan University, Wuhan, China; ^2^ Department of Surgery, Second Affiliated Hospital, Zhejiang University School of Medicine, Hangzhou, China; ^3^ Department of Biochemistry and Molecular Chemistry, School of Basic Medicial Sciences, Wuhan University, Wuhan, China

**Keywords:** liver cancer, tumor suppressor, lncRNA, mRNA stabilizing protein

## Abstract

Long non-coding RNAs (lncRNAs) have been identified as critical players in multiple cancers and lncRNAs are tightly linked to cancer progression. However, only little amount of lncRNAs have been identified to participate in the molecular mechanisms of the progression of hepatocellular carcinoma. In this study, we found that lncRNA-AK058003 is down-regulated in hepatocellular carcinoma tissues and it is associated with the relapse and metastasis of the cancer. Furthermore, lncRNA-AK058003 acts as a tumor suppressor, suppressing hepatocellular carcinoma cell proliferation and metastasis *in vitro* and *in vivo*. lncRNA-AK058003 can reduce mRNA stabilizing protein HuR, which results in the inhibition of the expression of γ-synuclein. In addition, a bioinformatics study indicated that γ-synuclein is a target of miR-15a. To verify whether lncRNA-AK058003 plays a role in miR-15a-mediated inhibition of γ-synuclein, we demonstrated that lncRNA-AK058003 is very likely to be a precursor of miR-15a. Collectively, lncRNA-AK058003 can reduce the expression of mRNA stabilizing protein HuR and act as a precursor of miR-15a to suppress γ-synuclein-mediated cell proliferation and the metastasis of hepatocellular carcinoma.

## INTRODUCTION

Hepatocellular carcinoma (HCC) has a poor prognosis and, after clinical diagnosis, is considered a deadly carcinoma that generally results in intra and extra hepatic metastasis. In spite of the improvement in prevention and treatment, HCC is still in the second leading cause of death worldwide [1]. Although previous studies have reported many genes involved in HCC, the molecular mechanisms remain unclear. Thus, a better understanding of the pathophysiological mechanisms of HCC is critical for uncovering molecular markers and developing therapeutic strategies.

Long non-coding RNAs (lncRNAs) are defined as transcripts with no evident protein coding function, and they are composed of more than 200 nucleotides and possess a 5′ cap and 3′poly-A tail [2]. LncRNAs have attracted great attention around the world and have been extensively investigated in recent years. The lncRNAs play an important role in the regulation of gene expression in normal physical conditions. However, the dysregulation of lncRNAs have been shown to be involved in the genesis, development and drug-resistance of many different cancers [3–5].

Rising evidence indicated that the aberrant expressions of lncRNAs are associated with tumor progression or metastasis. For example, LINC00628 in gastric cancer [6], H19 in lung cancer cells [7], 12-lncRNA in breast cancer [8], UCA1in bladder cancer [9] and 6-lncRNA in diffuse large-B-cell lymphoma [10]. Research on lncRNAs may not only help to elucidate the mechanisms of tumorigenesis and cancer aggression, but may also provide highly valuable targets for the diagnosis and therapy of cancer. A previous study suggested that lncRNA-AK058003 can be upregulated by hypoxia in gastric cancer (GC) and facilitate GC metastasis by targeting the γ-synuclein gene (SNCG) [11]. Furthermore, another publication confirmed that unregulated lncRNA-AK058003 significantly promotes the growth, invasion and migration of breast cancer via activating the SNCG [12]. However, whether lncRNA-AK058003 contributes to the prognosis of HCC and its functional mechanisms needs further exploration.

In this study, we investigated the expression level and biological function of lncRNA-AK058003 in HCC. To our surprise, the data indicated that expression of lncRNA-AK058003 was remarkably down-regulated in the liver cancer tissues. Furthermore, lncRNA-AK058003 could regulate cell growth and metastasis both *in vitro* and *in vivo*. In addition, lncRNA-AK058003 was an indirect transcriptional target of the SNCG by binding to HuR or miR-15a. Finally, we aimed to validate the functional role of lncRNA-AK058003, which acts as a tumor suppressor in HCC tumorigenicity.

## RESULTS

### LncRNA-AK058003 expression in HCC tissues and HCC patient survival

**Figure 1:**
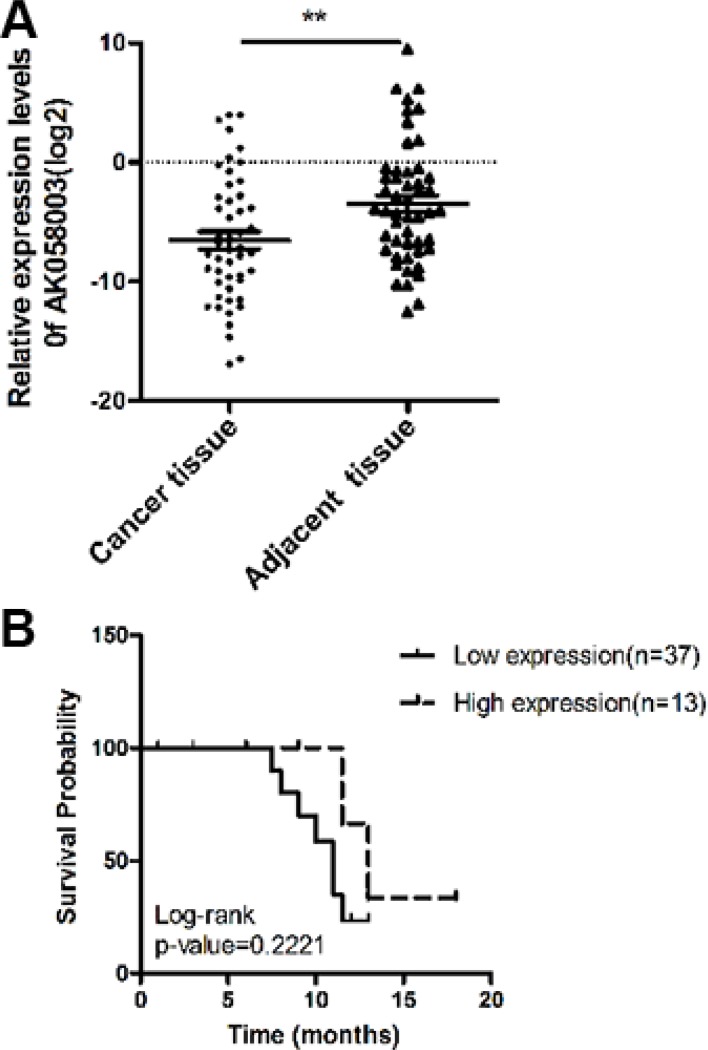
The association of lncRNA-AK058003 expression with HCC patient survival (**A**) LncRNA-AK058003 is significantly down-regulated in 50 human HCC tissues compared with adjacent tissues. Statistical differences were analyzed using a paired *t* test. (**B**) Overall survival of 50 liver cancer patients. There was no difference for patients with high lncRNA-AK058003 expression compared with low expression (*P* = 0.222, *n* = 50). Survival curves were compared using a long-rank test. **p < 0.01.

To investigate lncRNA-AK058003 expression levels between HCC and paired non-tumor samples, we performed quantitative real-time polymerase chain reaction (qRT-PCR) analysis on total RNA extracted from 50 pairs of tissues. The expression of lncRNA-AK058003 was remarkably down-regulated in HCC (*P* < 0.01), compared to their adjacent tissues (Figure 1A). This finding suggested that lncRNA-AK058003 expression differed from the two groups. In addition, we researched the correlation between lncRNA-AK058003 expression levels and clinicopathological features, including HBV infection (*n* = 36), hepatocirrhosis (*n* = 26), relapse and metastasis (*n* = 32). Unfortunately, there was no noteworthy correlation between lncRNA-AK058003 expression and age, gender, lump size, HBV infection, alpha-fetoprotein (AFP), hepatocirrhosis, or TNM stage (all *P* > 0.05). Nevertheless, it was remarkable that lncRNA-AK058003 down-regulation was prominently associated with relapse and metastasis (*P* = 0.027; Table 1). These data suggested that lncRNA-AK058003 may play a vital role in hepatic carcinostasis.

**Table 1:** Clinical characteristics of 50 HCC studied cohort according to AK058003 expression levels

Clinicopathological Factors	Frequency	AK058003		χ^2^test	*P* Value
		Low	High		
Age (year)				0.402	0.526
< 55	27	19	8		
≥ 55	23	18	5		
Gender				0.439	0.704
Female	11	9	2		
Male	39	28	11		
Size (cm)				0.124	0.724
< 5	21	15	6		
≥ 5	29	22	7		
HBV infection				1.621	0.203
Positive	36	25	11		
Negative	14	13	2		
AFP (ng/ml)				0.691	0.406
< 400	28	22	6		
≥ 400	22	15	7		
TNM stage				0.770	0.380
I/II	18	12	6		
III/IV	32	25	7		
Hepatocirrhosis				0.241	0.624
Positive	26	20	6		
Negative	24	17	7		
Relapse and metastasis				4.874	0.027*
Positive	32	27	5		
Negative	18	10	8		

Abbreviations: AFP, alpha-fetoprotein; TNM, tumor-node metastasis.

*Statistically significant (*P* < 0.05).

To further explore the relationship between lncRNA-AK058003 expression levels and the overall survival of HCC patients, the Kaplan-Meier and log-rank tests identified that the expression levels of lncRNA-AK058003 were less significantly associated with HCC patients’ prognosis (*P* = 0.222; Figure 1B). In addition, Cox regression analysis showed that HBV infection (95% CI, 0.005–1.111, *P* = 0.059) might potentially be prognostic factors in HCC (Table 2).

**Table 2:** Evaluate the prognosis factors in 50 HCC patients by cox regression analysis

Variables	Univariate analysis		
	Hazard ratio	95% CI	*P* value
Age	0.7341	0.1888–2.854	0.6555
Gender	1.959	0.3698–10.38	0.4292
Tumor size	0.5618	0.1335–2.365	0.4318
HBV infection	0.07573	0.005164–1.111	0.0596
AFP	0.4207	0.1053–1.681	0.2960
TNM stage	0.9475	0.2255–3.982	0.9413
Hepatocirrhosis	0.5407	0.1393–2.098	0.3741
Relapse and metastasis	1.767	0.4083–7.643	0.4464
LncRNA-AK058003	2.433	0.5838–10.14	0.2221

### LncRNA-AK058003 inhibits HCC cell proliferation

**Figure 2:**
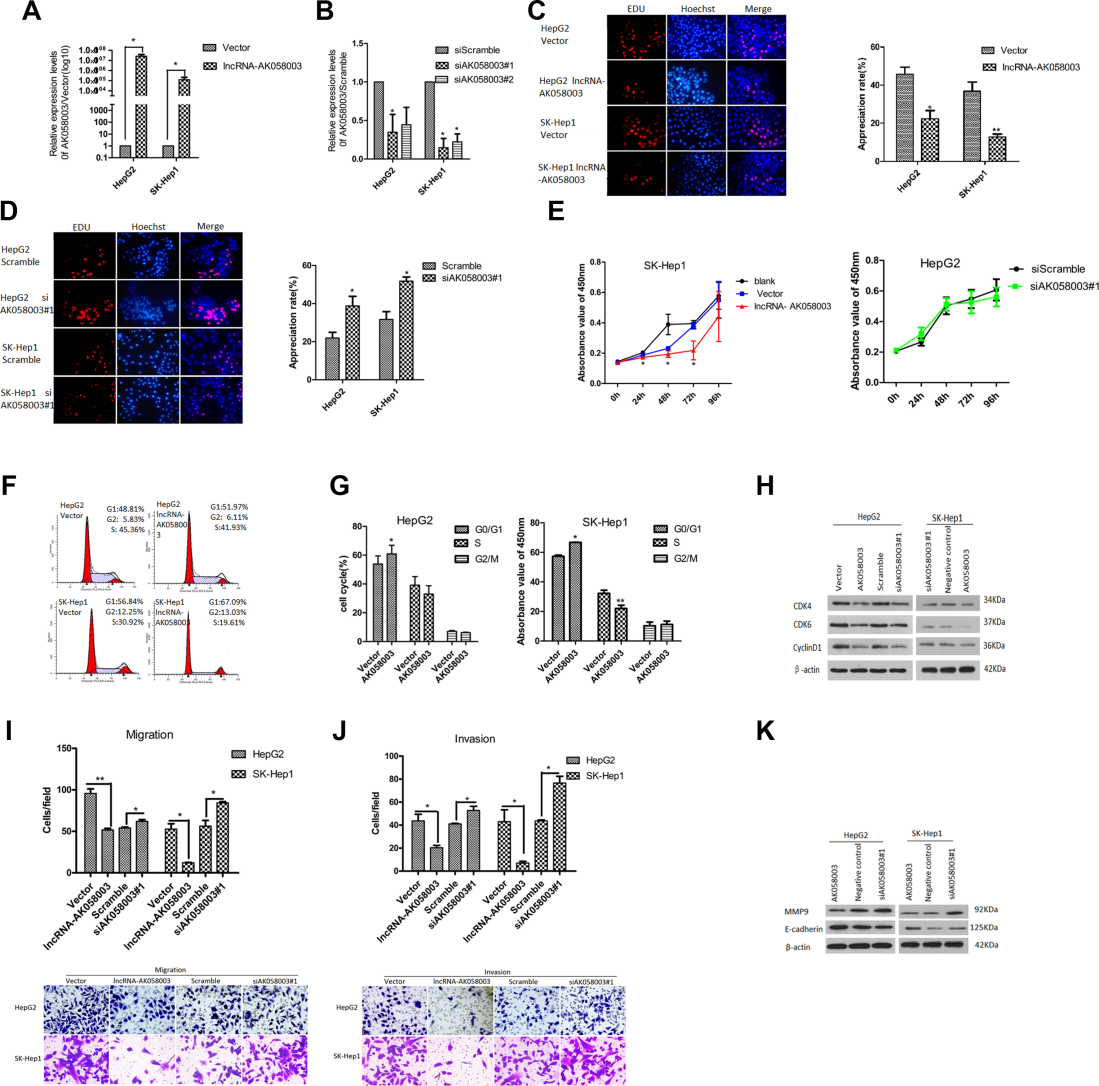
LncRNA-AK058003 inhibited cell proliferation, migration and invasion processes in vitro (**A**) HepG2 and SK-HeP1 stably over-expressing lncRNA-AK058003 cell lines were showed to have significantly increased expression levels of lncRNA-AK058003 compared to vector controls. (**B**) siRNA mediated knock-down of lncRNA-AK058003 (siAK058003) in HepG2 and SK-HeP1 cell lines were decreased significantly compared to scramble controls. (**C**) LncRNA-AK058003 up-regulated and (**D**). lncRNA-AK058003 down-regulated HepG2 and SK-HeP1 cell lines were seeded into 96 well plates and cell proliferation was assessed by EdU immunofluorescence staining. The proliferation ability value is in red. Original magnification, ×400. The barplot on the right shows the percentage of EdU-positive nuclei, indicating lncRNA-AK058003 notably restrained cell multiplication. (**E**) CCK8 assays showed that cell proliferation was suppressed by lncRNA-AK058003 overexpression in SK-HeP1 cells. The right curves show no significant difference in lncRNA-AK058003 down-regulated HepG2 cells. (**F**, **G**) FACS analysis showing a significant increase in the G1 phase in HepG2 and SK-HeP1 cells overexpressing lncRNA-AK058003. (**H**) The total protein expression of CDK4, CDK6, and cyclinD1 were detected by western blot analysis. Representative images of (**I**) transwell migration and (**J**) invasion assays in up-regulated and siRNA mediated knock-down of lncRNA-AK058003. Original magnification, × 400. (**K**) The metastasis related proteins (MMP9, E-cadherin) were performed in lncRNA-AK058003-overexpressing and lncRNA-AK058003- down-regulating HepG2 and SK-HeP1 cells by Western blot analysis. Data are shown as the mean ± S.E.M. based on three independent experiments. *p < 0.05, **p < 0.01, ***p < 0.001.

The frequent down-regulation of lncRNA-AK058003 in HCC specimens suggested that lncRNA-AK058003 may be a tumor suppressor gene in HCC. To investigate this conjecture, the biological significance of lncRNA-AK058003 expression was confirmed in two liver cancer cell lines (HepG2 and SK-HeP1). Gain-of-function and loss-of-function measurements were performed in subsequent studies. To investigate the role of lncRNA-AK058003 in HCC cell proliferation, EdU immunofluorescence staining and cell counting kit-8 (CCK8) were performed in lncRNA-AK058003 overexpressing cells (Figure 2A) and lncRNA-AK058003 down-regulated cells (Figure 2B). The EdU immunofluorescence staining assays (Figure 2C and 2D) demonstrated that lncRNA-AK058003 overexpressing cells (HepG2 and SK-HeP1) caused a remarkable decrease in EdU positive cells compared to the vector control group (Figure 2C). On the other hand, lncRNA-AK058003 down-regulated cells (HepG2 and SK-HeP1) were significantly increased in comparison to the scramble group (Figure 2D). In addition, CCK8 assays were applied after transfection at 0 h, 24 h, 48 h, 72 h and 96 h, respectively and the cell proliferation curves were drawn (Figure 2E and Supplementary Figure S1A, S1B). The CCK8 assay results were inconsistent with the EdU immunofluorescence staining results, except the siRNA-mediated lncRNA-AK058003 proliferation curve was not significantly different from the controls (Figure 2E and Supplementary Figure S1B).

To gain more insights into the role of lncRNA-AK058003 in HCC proliferation, we detected the differences in cell-cycles after lncRNA-AK058003 was up-regulated by FACS (Figure 2F). The results showed that the G0/G1 phase was prolonged in lncRNA-AK058003 up-regulated HepG2 and SK-HeP1 cells (Figure 2G). Consistent with flow cytometry data, the expression of cell-cycle checkpoint proteins such as cyclin-dependent kinase 4 (CDK4), cyclin-dependent kinase 6 (CDK6) and cyclinD1 were detected by western blot (Figure 2H). It showed that CDK4, CDK6 and cyclinD1 were markedly reduced in HepG2 and SK-HeP1 cells with overexpressed lncRNA-AK058003. Unfortunately, there was no significant difference in the expression of cell-cycle checkpoint proteins between lncRNA-AK058003 down-regulated HCC cells and the control group. Taken together, these results showed that lncRNA-AK058003 affects HCC cell growth *in vitro*.

### LncRNA-AK058003 inhibits HCC cell migration and invasion

We further assessed the function of lncRNA-AK058003 on cell metastasis, which is a key determinant of malignant progression. For transwell migration assays, overexpression of lncRNA-AK058003 in HepG2 and SK-HeP1 significantly reduced the number of migrating cells (Figure 2I). Next, knock-down of lncRNA-AK058003 in HepG2 and SK-HeP1 remarkably increased the effect of the migration (Figure 2I). In the meantime, we also performed gain-of-function and loss-of-function measurements of lncRNA-AK058003 in transwell invasion assays. The transwell invasion results were consistent with those of transwell migration assays (Figure 2J).

Additionally, analysis of the expression of metastasis protein markers such as MMP9 and E-cadherin by western blotting assays (Figure 2K) also confirmed the results above. These results suggested a functional role for lncRNA-AK058003 in HCC cell migration and invasion.

### LncRNA-AK058003 up-regulation inhibits HCC cell proliferation and metastasis *in vivo*

**Figure 3:**
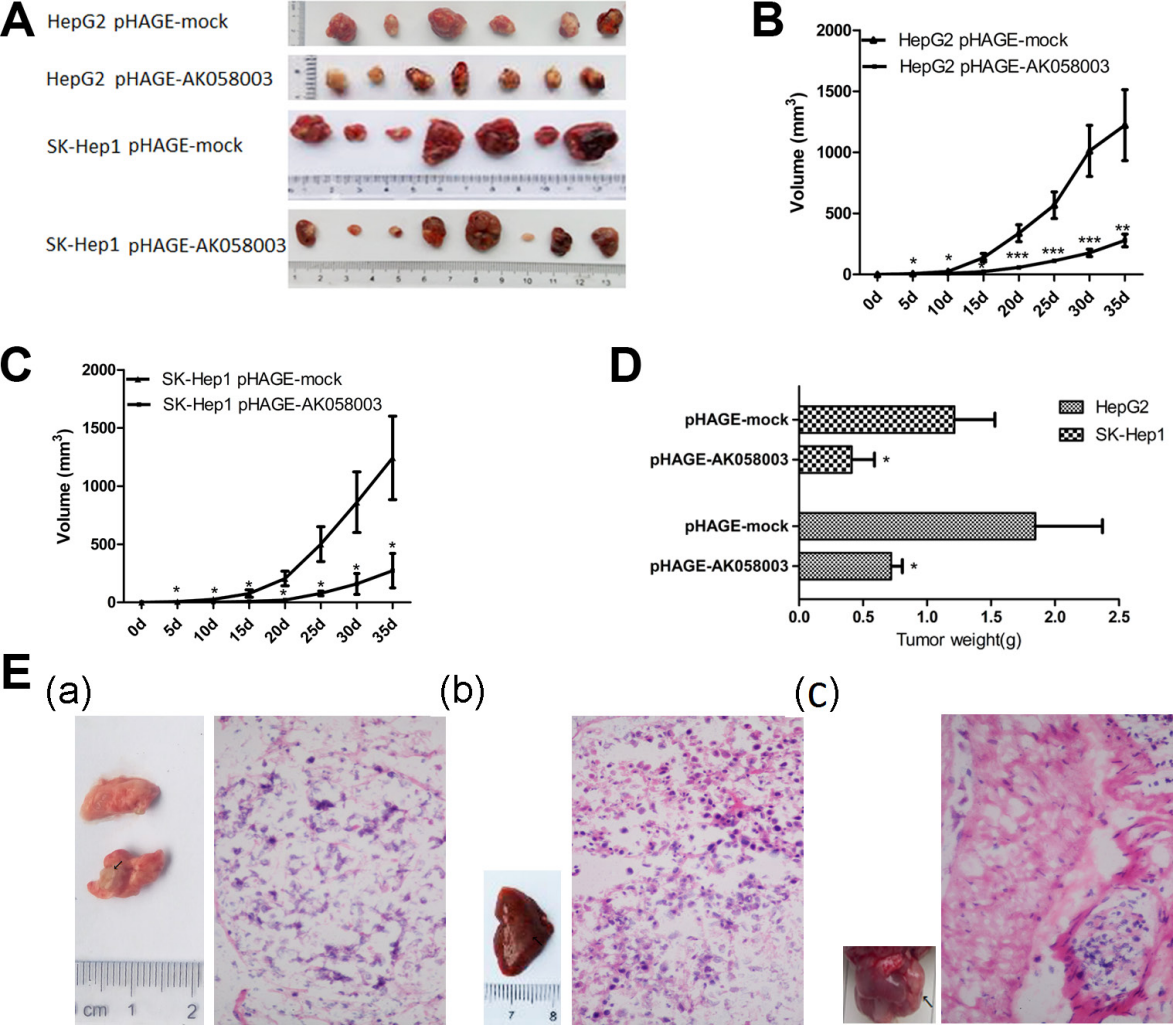
LncRNA-AK058003 suppressed tumor growth and metastasis *in vivo* (**A**) Photographs of tumors that were established to have lncRNA-AK058003-up-regulated HepG2 or with lncRNA-AK058003 -up-regulated SK-HeP1 cells in HCC xenograft-transplanted nude mouse tumor models. (**B**, **C**) Effects of lncRNA-AK058003 expression in HCC nude mouse tumor models are shown. (**D**) The effects of ectopic lncRNA-AK058003 expression in average tumor weights of nude mouse models. (**E**) These representative pictures show the metastasis in each group at 35 days after tail vein injection. (a) Pulmonary, (b) hepatic and (c) anogenital metastasis were detected in pHAGE-mock groups. The right images of metastasis tissues displayed pathomorphological changes by H&E staining. The arrows represent metastastic foci. Data are shown as the mean ± S.E.M. *p < 0.05, **p < 0.01,***p < 0.001.

LncRNA-AK058003 suppressed the proliferation, migration and invasion of HCC cells *in vitro*. To clarify the effects of lncRNA-AK058003 on cancer biological functions *in vivo*, HepG2 and SK-HeP1 cells stably overexpressing lncRNA-AK058003, or respective control cells, were injected into BALB/c nude mice. As a tumor proliferation model, stably up-regulated pHAGE-AK058003 and pHAGE-mock were injected into the left armpit of BALB/c nude mice. As shown in Figure 3A, and 3C, tumor growth in the pHAGE-AK058003 group was inhibited significantly compared with that of tumors formed from pHAGE-mock xenografts after 5 weeks: 299.6 ± 48.9 mm^3^ vs 1,335.9 ± 305.9 mm^3^ of HepG2 cells (*P* < 0.01); 272.4 ± 148.3 mm^3^ vs 1,242.8 ± 359.6 mm^3^ of SK-HeP1 cells (*P* < 0.05), respectively (Figure 3B and 3C). Up to 35 days after injection, the average tumor weight in the pHAGE-AK058003 group was significantly lower than the pHAGE-mock group (Figure 3D).

As a tumor metastasis model, a dose of (1 × 10^6^ cells) of stably overexpressing pHAGE-AK058003 cells and pHAGE-mock cells were injected into the tail vein of BALB/c nude mice. As shown in Table 3, tumor metastasis in pHAGE-mock cells was obviously higher than that in pHAGE-AK058003 cells. We further analyzed the metastatic tumors by H&E staining (Figure 3E). Taken together, these results supported lncRNA-AK058003 playing an inhibitory role in HCC growth and metastasis *in vivo*.

**Table 3:** The incidence of metastasis in nude mice after tail vein injection with tumor cells

Group	Lung metastasis	Abdominal cavity metastasis	Anogenital metastasis
pHAGE-mock	5/14	4/14	6/14
pHAGE-AK058003	0/15	1/15	0/15
P (χ^2^ test)	0.017*	0.169	0.006^*^

Note: pHAGE-mock represent blank control group of pHAGE carriers; pHAGE-AK058003 represent stably overexpression of lncRNA-AK058003.

*Statistically significant (*P* < 0.05).

### Association of LncRNA-AK058003 and SNCG

**Figure 4:**
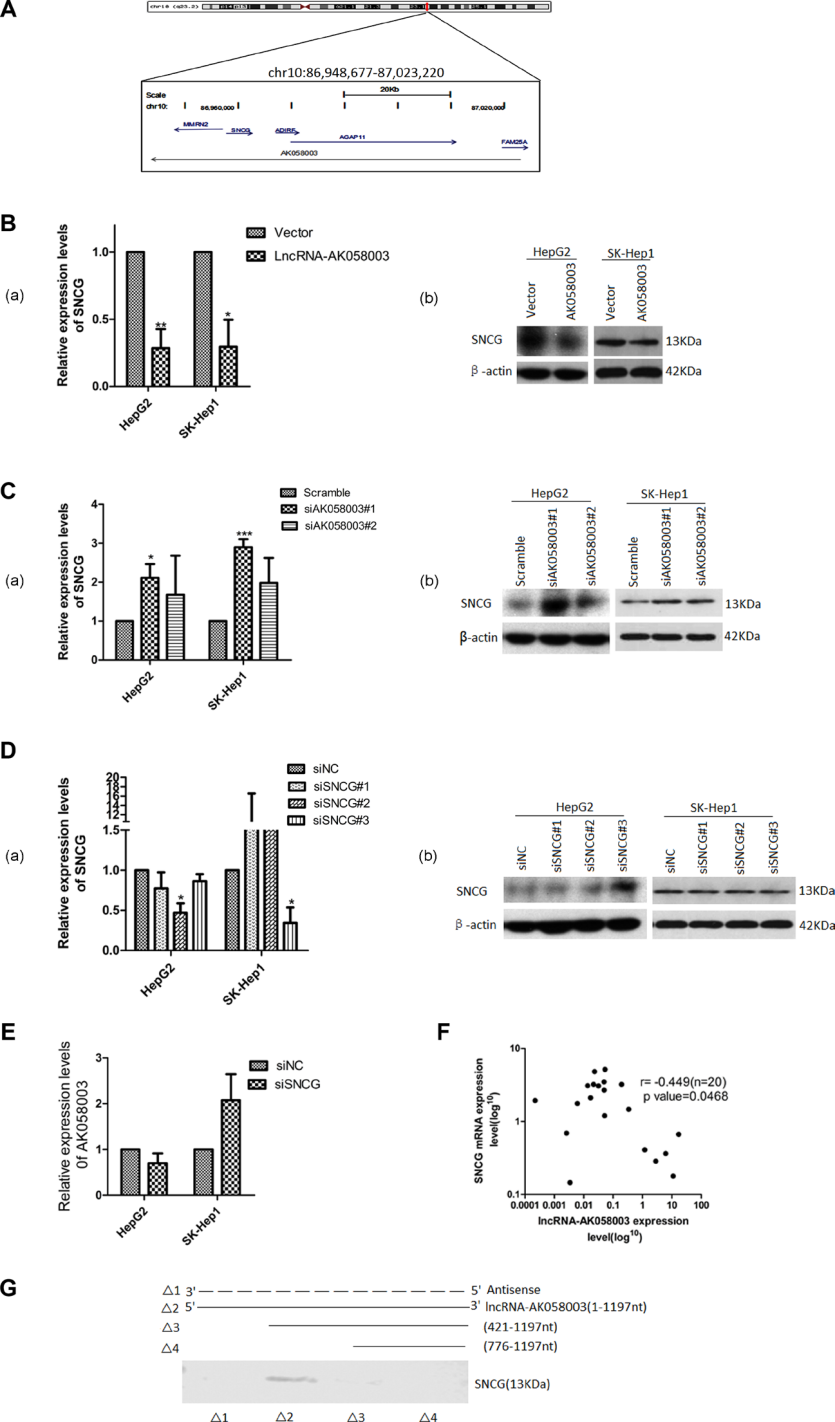
Association of lncRNA-AK058003 and SNCG (**A**) Schematic representation of the mRNA genes located around lncRNA-AK058003. Genes in blue represent mRNA genes, genes in gray represent non-coding RNA. (**B**) HepG2 and SK-HeP1 overexpressing lncRNA-AK058003 cell lines show remarkably reduced expression levels of SNCG than vector controls for both mRNA and protein levels. (**C**) siRNA mediated knock-down sequence-1 of lncRNA-AK058003 in HepG2 and SK-HeP1 cell lines show significantly increased mRNA and protein levels of SNCG than the scramble controls. (**D**) siRNA mediated knock-down of SNCG (siSNCG) in HepG2 and SK-HeP1 cells. Western blot and qRT-PCR revealed that SNCG expression levels were significantly reduced in HepG2 cells transfected with SNCG sequence-2 and in SK-HeP1 transfected with SNCG sequence-3. (**E**) LncRNA-AK058003 expression was not different between the siSNCG group and control group HCC cells. (**F**) Scatter diagram revealed a negative correlation between lncRNA-AK058003 and SNCG in 20 pairs of HCC tissues by qRT-PCR (*r* = −0.449, *P* = 0.047). (**G**) Biotinylated RNAs corresponding to different fragments of lncRNA-AK058003 or its sense sequences were incubated with SK-HeP1 cell lysates, captured with streptavidin beads and related SNCG was detected by western blot. Original magnification, ×400. Data are shown as the mean ± S.E.M. based on three independent experiments. *p < 0.05, **p < 0.01,***p < 0.001.

Recent studies have reported that lncRNA serves a crucial role in the regulation of the activity and functions of potential target genes [13, 14]. Hence, to clarify whether lncRNA-AK058003 suppresses HCC biobehavioral functions through this mechanism, we studied genomic information using bioinformatics software. The targets of lncRNA-AK058003 were predicted by searching the UCSC (http://genome.ucsc.edu/cgi-bin/hgGateway) and Ensemble Genome Browser (http://asia.ensembl.org/index.html?redirect = no). Five candidate targets were identified: SNCG, MMRN2, ADIRF, AGAP11, FAM25A (Figure 4A). The relationship between the target genes and lncRNA-AK058003 were examined in stably overexpressing lncRNA-AK058003 HCC cells by qRT-PCR and western blotting. As Figure 4B and 4C show, lncRNA-AK058003 can obviously inhibit mRNA expression of SNCG. Nevertheless, there was no difference in MMRN2, ADIRF, AGAP11 and FAM25A expression by qRT-PCR (data not shown). On the contrary, to explore regulation of the lncRNA-AK058003/SNCG gene axis in liver cancer cells, we down-regulated SNCG using siRNA (Figure 4D). Silencing of SNCG did not affect lncRNA-AK058003 expression (Figure 4E), suggesting that SNCG may be a key target of lncRNA-AK058003 in HCC cells.

To further evaluate whether lncRNA-AK058003 had an association with SNCG, we analyzed the expression of SNCG in 20 pairs of randomly selected HCC specimens using qRT-PCR (Figure 4F). In addition, we substantiated that SNCG expression levels were also up-regulated in cancer tissues compared to their adjacent samples, which was consistent with previous research [15, 16]. Spearman analyses conducted using qRT-PCR expression data from human samples showed that lncRNA-AK058003 and SNCG had a negative correlation score (*r* = −0.449, *P* = 0.047; Figure 4F). We next performed an RNA pull-down assay to identify the association between lncRNA-AK058003 and the SNCG. Deletion analysis identified a 421 nt region at the 5′ end of lncRNA-AK058003 that was required for the association with the SNCG (Figure 4G). Collectively, these data manifested that the SNCG is a target of lncRNA-AK058003.

### LncRNA-AK058003 interaction with the SNCG inhibits HCC growth and metastasis

**Figure 5:**
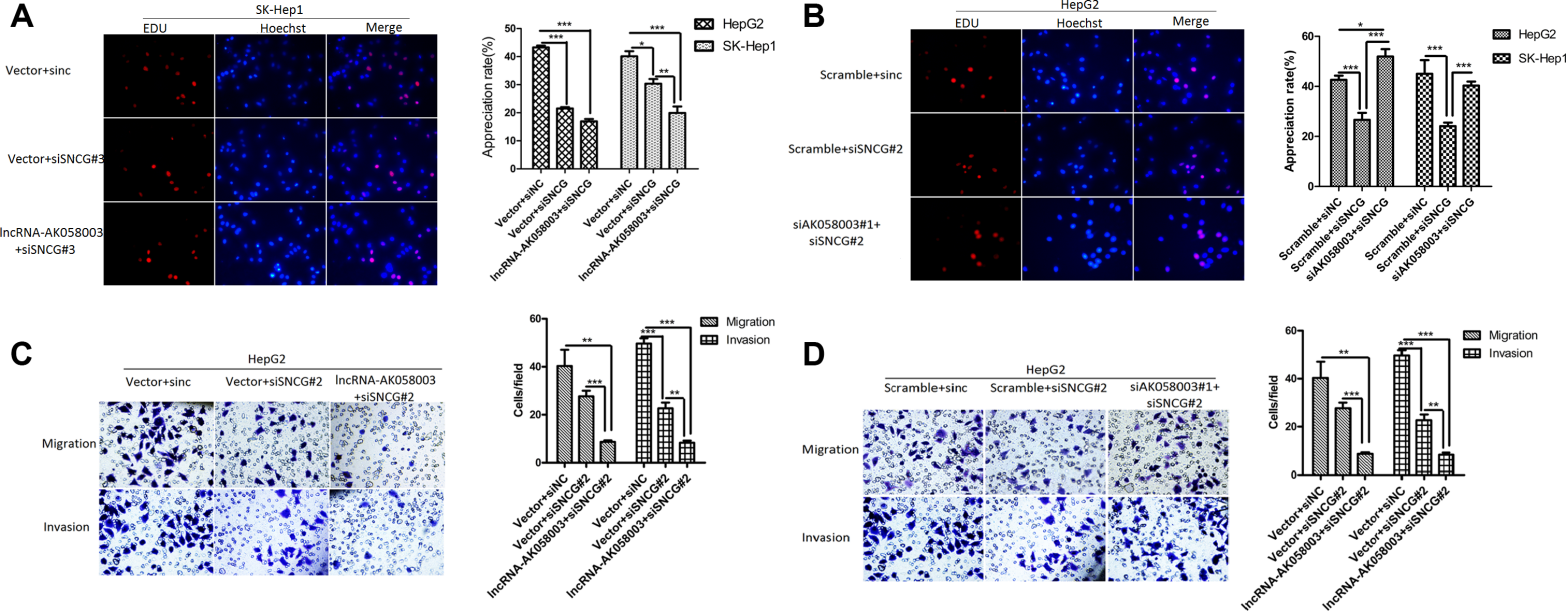
SNCG regulated by lncRNA-AK058003 in proliferation, migration and invasion of HCC Cells (**A**) LncRNA-AK058003 and siSNCG co-transfection in SK-HeP1 cells and (**B**) siAK058003#1 and siSNCG co-transfection in HepG2 cell lines were seeded into 96 well plates and cell proliferation was assessed by EdU immunofluorescence staining. Original magnification, ×400. The barplot on the right showed the percentage of EdU positive nuclei, indicating lncRNA-AK058003 restrained cell multiplication through suppressed SNCG. (**C**, **D)** Transwell migration and invasion assays of HepG2 cells were determined after co-transfection of lncRNA-AK058003 + siSNCG or siAK058003 + siSNCG. The graph on the right shows the positive cells per field. Original magnification, ×400. Data are shown as the mean ± S.E.M. based on three independent experiments.*p < 0.05, **p < 0.01, ***p < 0.001.

Previous studies have revealed that the SNCG acts as an oncogene in many cancers, including liver cancer, esophageal cancer, gastric cancer, colon cancer and cervical cancer [16, 18]. To further explore that the SNCG is a target gene of lncRNA-AK058003 factional in HCC proliferation, siRNA-mediated knock-down of the SNCG was transfected into stably overexpressing lncRNA-AK058003 cells, or siRNA that targeted the SNCG and down-regulating siRNA of lncRNA-AK058003 were co-transfected into HCC cells. As shown in Figure 5A, 5B and Supplementary Figure S4A, S4B, SNCG down-regulation significantly inhibited the proliferation of HCC cells. siSNCG was co-transfected into stably overexpressing lncRNA-AK058003 HCC cells, and the results showed that lncRNA-AK058003 up-regulated SK-HeP1 cells had obviously inhibited cell growth, except for HepG2 cells (Figure 5A and Supplementary Figure S4A). siSNCG and siAK058003 were co-transfected into HCC cells and the results indicated that lncRNA-AK058003 knock-down in SNCG down-regulated HCC cells could recover the effect of the SNCG (Figure 5B and Supplementary Figure S4B).

To further explore the SNCG as an indispensable gene of lncRNA-AK058003 in HCC metastasis, we performed the same co-transfection methods. As shown in Figure 5C and Supplementary Figure S4C, co-transfecting siSNCG into stably overexpressing lncRNA-AK058003 HCC cells showed that HCC cell migration and invasion was obviously decreased. In addition, siSNCG and siAK058003 were co-transfected into HCC cells and the results indicated that lncRNA-AK058003 knock-down in SNCG down-regulated HCC cells rescued the effect of the SNCG (Figure 5D and Supplementary Figure S4D). Collectively, these results showed that SNCG expression is reduced by lncRNA-AK058003 and this mediates HCC proliferation and metastasis.

### LncRNA-AK058003 binds HuR or acts as a precursor of miR-15a to regulate SNCG expression

**Figure 6:**
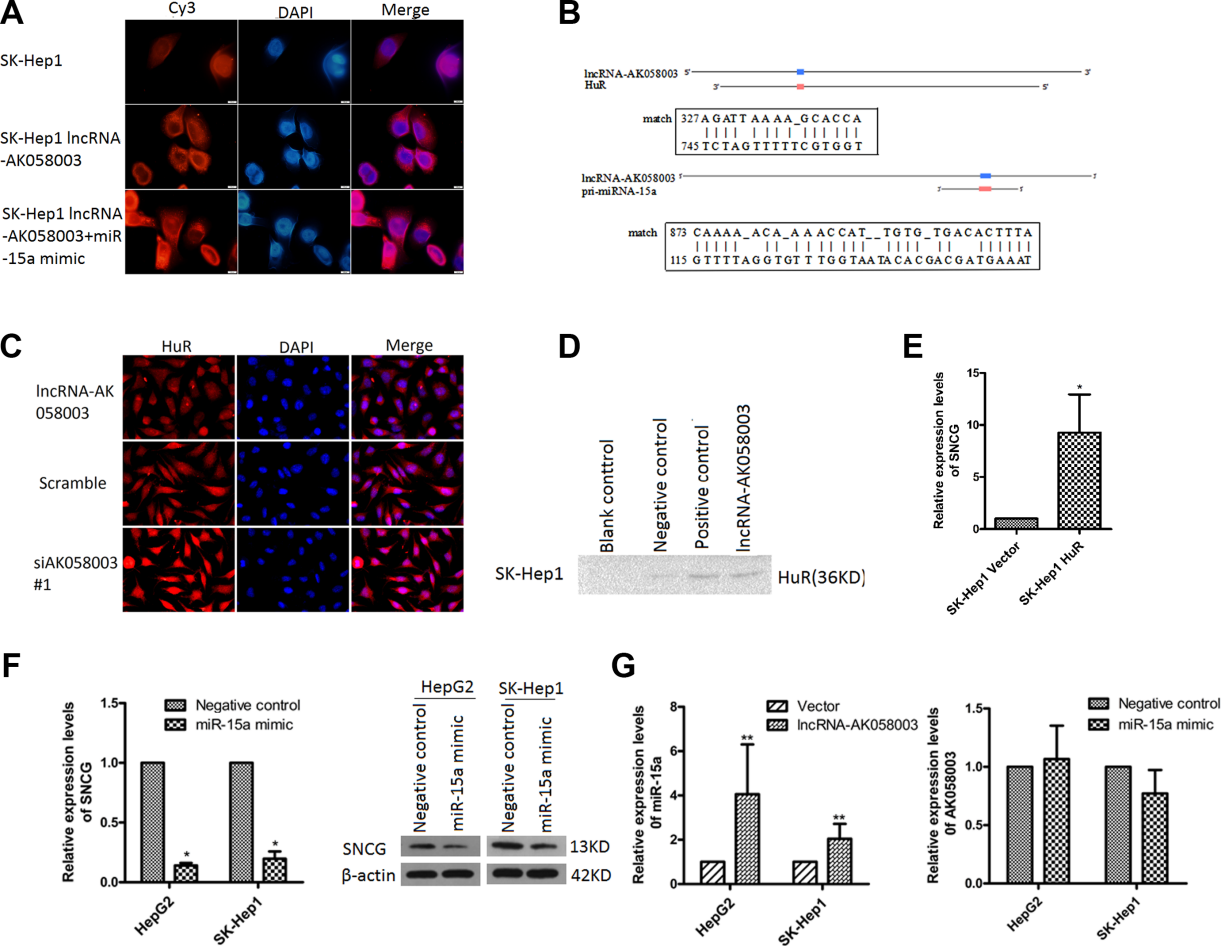
LncRNA-AK058003 binding with HuR or acts as a precursor of miR-15a to regulate SNCG expression (**A**) FISH detection of lncRNA-AK058003. Fixed SK-Hep1 cells with DAPI-stained nuclei (blue) were probed simultaneously for lncRNA-AK058003.Original magnification, ×1000. (**B**) The miR-15a target sites and HuR target sites in the sequence of lncRNA-AK058003, predicted by Clone Manager and Blast software. (**C**) The location and expression of HuR was detected by immunofluorescence staining after lncRNA-AK058003 overexpression and knockdown in HepG2 cells. (**D**) Biotinylated RNA of lncRNA-AK058003 or blank control (contain RIPA), negative control or positive control were incubated with SK-HeP1 cell lysates, captured with streptavidin beads and related HuR was detected by western blotting. (**E**) qRT-PCR showed that SNCG expression levels increased with overexpressing HuR. (**F**) Western blotting analysis and qRT-PCR showed that SNCG expression levels decreased with overexpression of miR-15a. (**G**) Increased miR-15a expression in HepG2 and SK-HeP1 cells overexpressing lncRNA-AK058003, The graph on the right shows that lncRNA-AK058003 expression levels in HepG2 and SK-HeP1 cells were not significantly correlated with miR-15a.Data are shown as the mean ± S.E.M. based on three independent experiments.*p < 0.05, **p < 0.01,***p < 0.001.

To investigate how lncRNA-AK058003 regulated the expression of the SNCG, we first studied lncRNA-AK058003 localization. Using fluorescence *in situ* hybridization (FISH) analysis, we found that lncRNA-AK058003 was located in the nucleus and cytoplasm of HepG2 and SK-HeP1 cells, especially in the cytoplasm (Figure 6A and Supplementary Figure S5A). As is known, cytoplasmic lncRNAs modulate gene transcription by interaction with RNA-binding proteins [19], or acts as a ceRNA in regulating the accumulation of miRNA and, in turn, affects its targets [20–23]. StarBase2.0 was used to predict and select miRNAs that interacted with the SNCG (Supplementary Table S1). Among these miRNA candidates, we found that miR-15a directly binds to the SNCG [24]. We performed bioinformatics analysis to identify the homologous sequences of lncRNA-AK058003, HuR and miR-15a. As shown in Figure 6B, HuR and miR-15a may act as potential target genes. Immunofluorescence (Figure 6C) and qRT-PCR (Supplementary Figure S5B, S5C) showed that the expression of HuR had a negative correlation with lncRNA-AK058003. Furthermore, an RNA pull-down assay also showed an association between lncRNA-AK058003 and HuR (Figure 6D). As shown in Figure 6E, overexpression of HuR (Supplementary Figure S5D) could increase mRNA expression of the SNCG.

Consistent with the above findings, overexpression of miR-15a (Supplementary Figure S5E) obviously suppressed the mRNA and protein levels of the SNCG (Figure 6F). To verify whether lncRNA-AK058003 plays a role in miR-15a-mediated inhibition of the SNCG, we detected the RNA level of lncRNA-AK058003 in miR-15a overexpressing HCC cells (Figure 6A, 6G and Supplementary Figure S5A). Interestingly, up-regulated lncRNA-AK058003 obviously increased miR-15a expression (Figure 6G), while lncRNA-AK058003 was not affected by miR-15a. Taken together, these data showed that lncRNA-AK058003 might act as a precursor of miR-15a in SNCG-mediated proliferation and metastasis of HCC.

## DISCUSSION

In the current study, we found that lncRNA-AK058003 is down-regulated in HCC specimens, and we identified its expression is associated with relapse and metastasis. Thus, we believe that lncRNA-AK058003 might have an important biological function in HCC. The failure to identify a correlation with lncRNA-AK058003 expression levels and overall survival of HCC patients (*P* = 0.222) may be attributed to the short time of follow-up (12.30 ± 0.80) and few samples. Further evidence in this patient cohort with a prolonged follow-up should be undertaken.

**Figure 7:**
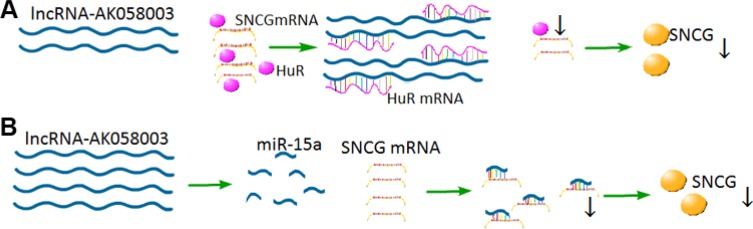
Proposed model for the regulation mechanism lncRNA-AK058003 in HCC cells (**A**) LncRNA-AK058003 shares response elements with HuR.Up-regulated lncRNA-AK058003 acts as a decoy target HuR, leading to decreased expression of HuR and SNCG. (**B**) LncRNA-AK058003 and miR-15a share some of the same sequences. Up-regulated lncRNA-AK058003 could increase the expression of miR-15a, which is an inhibitor of SNCG.

LncRNA-AK058003 is a 1,197 nt transcript that is located on chromosome 10q22 on the reverse strand with a weak ability for coding protein. To further investigate the mechanism of lncRNA-AK058003 in HCC cells, gain-of-function and loss-of-function measurements were conducted. We showed that lncRNA-AK058003 can inhibit HCC proliferation and metastasis both *in vitro* and *in vivo*, acting as a tumor suppressor and can be regarded as a prognostic indicator. Different from gastric cancer [11] and breast cancer [12], the expression of lncRNA-AK058003 may be associated with the tumor microenvironment and the position of the gene. We next looked for the molecular mechanism by which lncRNA-AK058003 regulates the biological behaviors of HCC. Recent studies have clarified that lncRNA typically binds special proteins for functional effects [25, 26]. An RNA pull-down assay indicated that lncRNA-AK058003 could combine with the SNCG. Thus, we tested the interaction of lncRNA-AK058003 with the SNCG in HCC growth and metastasis. The results showed that lncRNA-AK058003 could regulate the expression of SNCG in HCC proliferation and metastasis. However, the detailed regulatory molecular mechanisms need investigation. Previous research showed the function of lncRNA depended on its location. Most of the lncRNA exists in the nucleus, which means that it may regulate gene chromosome recombination, splicing and transcription. A portion of lncRNA is enriched in the cytoplasm and may regulate mRNA translation [27], mRNA stability [28] and protein localization [17]. In our study, a FISH assay revealed that lncRNA-AK058003 exists in the cell nucleus and cytoplasm, and is mainly located in the cytoplasm, which suggests that it might adjust gene transcription and translation. As is known, cytoplasmic lncRNA interacts with RNA-binding proteins to regulate gene transcription. An RNA-pull down assay demonstrated that lncRNA-AK058003 could bind to HuR, an RNA-binding protein. Overexpression of HuR could increase the mRNA expression of SNCG. Collectively, these results demonstrated that lncRNA-AK058003 inhibited HCC progression by suppressing the SNCG in a HuR-dependent manner (Figure 7A).

Moreover, the SNCG encodes a member of the synuclein family of proteins, which are believed to be involved in the pathogenesis of neurodegenerative diseases [29]. Previous work has shown that the SNCG is up-regulated in many cancer types, including HCC, esophageal cancer, gastric cancer, colon cancer, breast cancer, lung cancer, cervical cancer, prostate cancer and diffuse large-B-cell lymphoma [3–10]. Ping Li et al. [24] found that the SNCG contributes to the cell cycle and cell apoptosis in breast cancer, and that miR-15a directly targets the SNCG. Recently, the interaction between lncRNA and miRNA has inspired much interest. Thus, we extended the research to determine whether lncRNA-AK058003 regulates SNCG expression, indirectly targeted by miR-15a or not. Up-regulated lncRNA-AK058003 obviously increased miR-15a expression and they share some of the same sequences, suggesting that lncRNA-AK058003 may regulate SNCG-mediated HCC proliferation and metastasis as a precursor of miR-15a (Figure 7B).

In summary, our study showed that lncRNA-AK058003 is a suppressor gene that inhibits HCC cell proliferation and metastasis *in vitro* and *in vivo* in a HuR-SNCG-dependent manner. We describe a paradigm such that lncRNA-AK058003 might function as a HuR decoy and suppress HuR expression, affecting SNCG translation and stability. Further study of the signaling pathways influenced by lncRNA-AK058003 may provide more insight into potential novel strategies for the diagnosis and therapy of HCC.

## MATERIALS AND METHODS

### Patient specimens

Fifty pairs of HCC tissue samples and corresponding adjacent tissues were obtained from the Department of Surgery of Renmin Hospital of Wuhan University (Wuhan, China) from June 2014 to June 2016. All the related sample clinicopathological data were obtained from the department of Pathology and the Hepatology Outpatient Clinic and are summarized in Table 1. The employed criteria were from the AJCC TNM Classification system. Patients’ specimens were preserved in liquid nitrogen until RNA extraction. The median follow-up was 18 months. All human tissue samples were obtained with informed consent and approved by the Clinical Research Ethics Committee at the Renmin Hospital of Hubei Province.

### Cells culture and culture conditions

The human liver cancer cell lines, including HepG2 and SK-Hep1 were obtained from the Institute of Cell Biology, Procell (Wuhan, China). The human embryonic kidney 293T (HEK 293T) cells also purchased from the Procell company(Wuhan, China). Specifically, HepG2 and HEK 293T cells were cultivated with high glucose medium, DMEM (Hyclone, Logan, USA), while SK-Hep1 cells were maintained in MEM (Hyclone, Logan, USA), supplemented with 10% fetal bovine serum (Gibco, Life Technologies, USA) and 1% penicillin-streptomycin (Hyclone, Logan, USA). The culture medium was changed every 48 h.

### Lentiviral construction and cell transfection

To establish clones stably overexpressing lncRNA-AK058003, the lncRNA-AK058003 full-length (GenBank reference sequence GI:16554001) or an empty vector were cloned into the lentivector pHAGE-CMV-MCS-IRES-ZsGreen cDNA cloning and expression vector (Addgene, Cambridge, UK) between BamH I and Xho I sites. HEK 293T cells were cultured in 15 cm plates at 70–80% confluence before transfection using Calcium chloride (Sigma, Japan) reagent. After 24 h post-transfection, the mixture was replaced with complete DMEM. The virus medium was collected at 72 h and ultracentrifuged at 87,000 g for 2 h at 4°C. The HepG2 and SK-Hep1 cells infected with resuspended lentivirus were visualized by fluorescent microscopy (Olympus BX51). Two days after the lentivirus infection, puromycin was used to select stable clones.

Both the siRNAs and miRNA mimics were synthesized by the Ribobio Company (Guangzhou, China). The detailed target sequences are listed in Supplementary Table S2. The HepG2 and SK-Hep1 cells were seeded in a 6-well plate and maintained with complete medium without antibiotics. A transfection assay was practiced when the cell confluence reached about 70%. Oligonucleotide and plasmid transfections were conducted by Lipofectamine 2000 (Life Technologies, USA). After incubation for 6 h at 37°C, the original medium was replaced and harvested after incubation for another 48 h.

### Bioinformatics analysis

The location of lncRNA-AK058003 were found in UCSC (http://genome.ucsc.edu/cgi-bin/hgGateway) and Ensemble Genome Browser (http://asia.ensembl.org/index.html?redirect = no). The ORF prediction was measured in ORF finder (https://www.ncbi.nlm.nih.gov/orffinder/). The encoding protein ability of lncRNA was detected in Coding Potential Calculator (http://cpc.cbi.pku.edu.cn/) [30]. The miRNA target prediction lncRNA sequences were obtained from TargetScan website (http://www.targetscan.org/). For lncRNA predict miRNAs’ target genes, starbase v2.0 (http://starbase.sysu.edu.cn/), Clone Manager and Blast software (http://blast.ncbi.nlm.nih.gov/Blast.cgi) were applied. All primers were designed by Clone manager software and Blast.

### Quantitative real-time polymerase chain reaction

Total RNA of cells and tissues was extracted with Trizol Reagent (Life Technologies, USA) following the manufacturer's protocol. Then RNAs were reversely transcribed by RT-PCR Quick Master Mix Kit (TOYOBO, OSAKA, Japan). The qRT-PCR was performed in Bio-Rad CFX Manager 2.1 real-time PCR Systems (Bio-Rad, USA) using SYBR Green Mix Kit (Takara Bio, shiga, Japan). Glyceraldehyde-3-phosphate dehydrogenase (GAPDH), β-actin or U6 were used as internal controls. All the primers sequences are described in Supplementary Table S3 (Tianyihuiyuan, Beijing, China). The relative expression quantity of candidate genes were analyzed basing on the equation 2^−ΔΔCt^ method.

### Xenograft tumor model

Male athymic BALB/c nude mice (age, 2–4 weeks old) were purchased from the HFK Bioscience company (Beijing, China) and randomly divided into four groups. Both lncRNA-AK058003 stably overexpressing HepG2 or SK-Hep1 cells and control cells (2 × 10^6^ CP20) were implanted in the armpit area of nude BALB/c nude mice (Supplementary Figure S2). To assess the effects of lncRNA-AK058003 on tumor metastasis, treatment was initiated 1 week after tail vein injection (1 × 10^6^ CP20) of stably overexpressing lncRNA-AK058003 cells or control cells. The tumor growth and tumor volume were calculated using the formula: Volume = (maximum diameter × minimum diameter^2^)/2, every 5 days after implantation. All nude mice were sacrificed after 35 days, and the xenograft tumors were dissected and weighed. The samples were snap frozen in liquid nitrogen immediately. The animal experimentation handling was conducted in accordance with the Animal Care and Euthanasia in Animal Studies Committee at Wuhan University.

### Flow cytometry assay

The cell-cycle analysis was conducted with a fluorescence-activated cell sorting (FACS) Calibur flow cytometer (Becton-Dickinson, Franklin Lakes, NJ, USA). Cells (1 × 10^6^) were resuspended by 1ml staining buffer A and 10 μL reagent B (MultiScience Biotech, Hangzhou, China), according to the product's instructions. Finally, data analysis was performed using FCS express version 3 software (DeNovo Software, Los Angeles, CA, USA); the percentages of cells in G0/G1, S, and G2/M phase were counted and compared.

### Cell proliferation assay

The Cell Counting Kit-8 (CCK-8, Dojindo, Kyushu, Kumamoto, Japan) assay was used to examine cell proliferation according to the manufacturer's instructions. After 0 h, 24 h, 48 h, 72 h, and 96 h, CCK-8 was added to the wells and incubated for 2 h at 37°C. Next, the absorbance at 450 nm was detected with an automatic micro-plate reader (Bio-Rad, Hercules, CA, USA) with the purpose of plotting the growth curve. To visually observe the proliferative cells, an EdU incorporation experiment was conducted using an EdU immunofluorescence staining kit (Ribobio, Guangzhou, China). The cells were seeded into 96-well culture plates at a density of 4 × 10^3^ cells/well. EdU medium (1:1,000) was add to the wells and incubated at 37°C for 2 hours. Cells were cleaned by phosphate buffer solution (PBS; Hyclone, Logan, Utah, USA) before being fixed in 4% phosphate-buffered paraformaldehyde. Then cells were stained with fresh Apollo solution and the nucleus was stained with Hoechst33342. Finally, cells were observed with a microscope (Olympus BX51).

### Transwell chamber assay

Transwell migration and invasion assays were assessed with Transwell Chambers 3422 (Corning, Kennebunk, USA). For a migration assay, 3 × 10^4^ cells in 200 μL serum-free medium after transfection for 24 h were seeded in the upper compartment of the chamber. We added 600 μL complete medium containing 10% FBS were added into the lower chamber. For an invasion assay, the upper surface of polycarbonic membranes was covered with a 60 μL matrigel (Becton-Dickinson, Franklin Lakes, NJ, USA) and incubated for 2 h at 37°C. The subsequent operations were similar to the migration assay. After incubation for another 24 h, the cells on the upper surface were removed with cotton swabs. Then the cells that had invaded into the microporous membrane were washed three times with PBS and fixed with 4% paraformaldehyde solution for 30 min, air dried, and stained with 0.1% crystal violet (Google biotechnology, Wuhan, China) for 20 min. Finally, the cells were observed with a microscope (Olympus BX51) and images were captured as well.

### RNA pull-down assay

The RNA pull-down assay was performed as described previously [31, 32]. Briefly, biotin-labeled RNAs were *in vitro* transcribed with a RNA 3′ end desthiobiotinylation kit (Thermo Fisher scientific, USA) and purified with the RNeasy Mini kit (Qiagen, Inc., Valencia, CA). To detect the biotinylated RNA by dot blotting (Supplementary Figure S3), we followed the protocol of the Thermo Scientific Chemiluminesent Nucleic Acid Detection Module kit (Thermo Fisher Scientific, USA). Two milligrams of HCC cell protein extract were then mixed with 50 pmol of biotinylated RNA biotin-labeled RNAs, incubated with streptavidin magnetic beads and washed. Finally, the eluted proteins were detected on SDS-PAGE gels for western blot. The western blot in an RNA-pull down assay was performed with rabbit anti-SNCG (Abcam, 1:400), mouse anti-HuR (Santa, 1:500) and goat anti-IgG (H + L; Proteintech, 1:10,000).

### Fluorescence *in situ* hybridization (FISH) and immunofluorescence analysis

The FISH protocol was conducted as previously described [33]. The oligonucleotide probes and FISH kit were designed and purchased from RiboBio Company, Ltd (Guangzhou, China). The probes targeting lncRNA sequences were combined with the fluorophore Cy3. Briefly, before fixation, HepG2 and SK-Hep1 cells were cultured in 6-well plates with glass slides and grown 24 h at 37°C. Cells were fixed in 4% paraformaldehyde for 10 min at room temperature, washed with PBS thrice and permeabilized with 0.5% Triton x-100 in the plates. Before hybridization, the cells were pre-hybridized with pre-hybridization buffer containing 1% blocking solution for 30 min at 37°C. Then, the probes (2.5 μl, 20 μM) were hybridized in hybridization buffer at 37°C overnight. After hybridization, the cells were washed in wash buffer I at 42°C for 5 min thrice, in wash buffer II for 5 min at 42°C and then in wash buffer III at 42°C for 5 min. 4, 6-Diamidino-2-phenylindole (DAPI) was used to detect the nuclei signals.

For immunocytochemistry analysis, SK-Hep1 cells transfected with lncRNA-AK058003 or siAK058003 were cultured on glass slides. Cells were fixed in 4% paraformaldehyde for 15 min at room temperature and washed with PBS thrice in the plates. Slides were blocked with 5% bovine serum albumin (BSA) diluted in TBS-T solution for 30 min at room temperature. After first being incubated with rabbit anti-SNCG antibodies (Abcam, 1:400), and then with goat anti-rabbit IgG (Proteintech, 1:10000), the slides were mounted with DAPI-Fluoromount-G (Southern Biotech, SBA, Birmingham, AL) and observed with a microscope (Olympus BX51).

### Protein extraction and western-blot assay

The whole cell protein was lysed on ice in RIPA (Upstate Biotechnology, Charlottesville, VA) containing protease inhibitor cocktail (Calbiochem). Quantified proteins lysates were separated by a SDS-PAGE gel and transferred to polyvinylidene fluoride (PVDF) membranes (Bio-Rad, USA). Proteins bands were visualized using an Enhanced Chemiluminescence Detection Kit (Biofavor BiotechService, Wuhan, China) and exposed to X-ray films (Kodak,USA). The β-actin was used as the internal standard. The membrane were probed with the following antibodies: SNCG antibodies (Abcam, ab6169), CDK4 (Bioworld, BS6462), CDK6 (Bioss, bs-0568R), CyclinD1 (CST, 2978P), MMP9 (Bioworld, bs6893) and E-cadherin (ProteinTech, 20874-1-AP).

### Statistical analysis

All statistical analyses were performed using IBM SPSS20.0 statistics (IBM, USA). Measurement data analysis between two groups was conducted by using a Student's *t*-test, while comparison of multiple groups was conducted using one-way analysis of variance (ANOVA). In addition, a chi-squared test (χ^2^ test) and Fisher's exact test were adopted to analyze the enumerated data. Survival curves were performed using Kaplan-Meier analysis and log-rank tests. Correlations were determined with a Spearman's rank correlation analysis. *P*-values less than 0.05 were considered significant (*p < 0.05, **p < 0.01, ***p < 0.001). Data are presented as mean ± S.E.M. All graphs and curves were made with GraphPad Prism 5 (GraphPad Software, CA, USA).

## SUPPLEMENTARY MATERIALS FIGURES AND TABLES



## References

[1] Chen W, Zheng R, Baade PD, Zhang S, Zeng H, Bray F, Jemal A, Yu XQ, He J (2016). Cancer statistics in China, 2015. CA Cancer J Clin.

[2] Wei G, Luo H, Sun Y, Li J, Tian L, Liu W, Liu L, Luo J, He J, Chen R (2015). Transcriptome profiling of esophageal squamous cell carcinoma reveals a long noncoding RNA acting as a tumor suppressor. Oncotarget.

[3] Ma MZ, Zhang Y, Weng MZ, Wang SH, Hu Y, Hou ZY, Qin YY, Gong W, Zhang YJ, Kong X, Wang JD, Quan ZW (2016). Long Noncoding RNA GCASPC, a Target of miR-17-3p, Negatively Regulates Pyruvate Carboxylase-Dependent Cell Proliferation in Gallbladder Cancer. Cancer Res.

[4] Yuan SX, Wang J, Yang F, Tao QF, Zhang J, Wang LL, Yang Y, Liu H, Wang ZG, Xu QG, Fan J, Liu L, Sun SH (2016). Long noncoding RNA DANCR increases stemness features of hepatocellular carcinoma by derepression of CTNNB1. Hepatology.

[5] Zhou M, Wang X, Li J, Hao D, Wang Z, Shi H, Han L, Zhou H, Sun J (2015). Prioritizing candidate disease-related long non-coding RNAs by walking on the heterogeneous lncRNA and disease network. Mol Biosyst.

[6] Zhang ZZ, Zhao G, Zhuang C, Shen YY, Zhao WY, Xu J, Wang M, Wang CJ, Tu L, Cao H, Zhang ZG (2016). Long non-coding RNA LINC00628 functions as a gastric cancer suppressor via long-range modulating the expression of cell cycle related genes. Sci Rep.

[7] Barsyte-Lovejoy D, Lau SK, Boutros PC, Khosravi F, Jurisica I, Andrulis IL, Tsao MS, Penn LZ (2006). The c-Myc oncogene directly induces the H19 noncoding RNA by allele-specific binding to potentiate tumorigenesis. Cancer Res.

[8] Zhou M, Zhong L, Xu W, Sun Y, Zhang Z, Zhao H, Yang L, Sun J (2016). Discovery of potential prognostic long non-coding RNA biomarkers for predicting the risk of tumor recurrence of breast cancer patients. Sci Rep.

[9] Pan J, Li X, Wu W, Xue M, Hou H, Zhai W, Chen W (2016). Long non-coding RNA UCA1 promotes cisplatin/gemcitabine resistance through CREB modulating miR-196a-5p in bladder cancer cells. Cancer Lett.

[10] Sun J, Cheng L, Shi H, Zhang Z, Zhao H, Wang Z, Zhou M (2016). A potential panel of six-long non-coding RNA signature to improve survival prediction of diffuse large-B-cell lymphoma. Sci Rep.

[11] Wang Y, Liu X, Zhang H, Sun L, Zhou Y, Jin H, Zhang H, Liu J, Guo H, Nie Y, Wu K, Fan D, Zhang H (2014). Hypoxia-inducible lncRNA-AK058003 promotes gastric cancer metastasis by targeting gamma-synuclein. Neoplasia.

[12] He K, Wang P (2015). Unregulated long non-coding RNA-AK058003 promotes the proliferation, invasion and metastasis of breast cancer by regulating the expression levels of the gamma-synuclein gene. Exp Ther Med.

[13] Khalil AM, Guttman M, Huarte M, Garber M, Raj A, D Rivea Morales, Thomas K, Presser A, Bernstein BE, van Oudenaarden A, Regev A, Lander ES, Rinn JL (2009). Many human large intergenic noncoding RNAs associate with chromatin-modifying complexes and affect gene expression. Proc Natl Acad Sci USA.

[14] Huarte M, Guttman M, Feldser D, Garber M, Koziol MJ, Kenzelmann-Broz D, Khalil AM, Zuk O, Amit I, Rabani M, Attardi LD, Regev A, Lander ES (2010). A large intergenic noncoding RNA induced by p53 mediates global gene repression in the p53 response. Cell.

[15] Liu C, Guo J, Qu L, Bing D, Meng L, Wu J, Shou C (2008). Applications of novel monoclonal antibodies specific for synuclein-gamma in evaluating its levels in sera and cancer tissues from colorectal cancer patients. Cancer Lett.

[16] Liu H, Liu W, Wu Y, Zhou Y, Xue R, Luo C, Wang L, Zhao W, Jiang JD, Liu J (2005). Loss of epigenetic control of synuclein-gamma gene as a molecular indicator of metastasis in a wide range of human cancers. Cancer Res.

[17] Jiang Y, Liu YE, Goldberg ID, Shi YE (2004). Gamma synuclein, a novel heat-shock protein-associated chaperone, stimulates ligand-dependent estrogen receptor alpha signaling and mammary tumorigenesis. Cancer Res.

[18] Raposo TP, Pires I, Prada J, Queiroga FL, Argyle DJ (2016). Exploring new biomarkers in the tumour microenvironment of canine inflammatory mammary tumours. Vet Comp Oncol.

[19] Li Z, Chao TC, Chang KY, Lin N, Patil VS, Shimizu C, Head SR, Burns JC, Rana TM (2014). The long noncoding RNA THRIL regulates TNFα expression through its interaction with hnRNPL. Proc Natl Acad Sci USA.

[20] Poliseno L, Salmena L, Zhang J, Carver B, Haveman WJ, Pandolfi PP (2010). A coding-independent function of gene and pseudogene mRNAs regulates tumour biology. Nature.

[21] Jeyapalan Z, Deng Z, Shatseva T, Fang L, He C, Yang BB (2011). Expression of CD44 3′-untranslated region regulates endogenous microRNA functions in tumorigenesis and angiogenesis. Nucleic Acids Res.

[22] Cesana M, Cacchiarelli D, Legnini I, Santini T, Sthandier O, Chinappi M, Tramontano A, Bozzoni I (2011). A long noncoding RNA controls muscle differentiation by functioning as a competing endogenous RNA. Cell.

[23] Kumar MS, Armenteros-monterroso E, East P, Chakravorty P, Matthews N, Winslow MM, Downward J (2014). HMGA2 functions as a competing endogenous RNA to promote lung cancer progression. Nature.

[24] Li P, Xie XB, Chen Q, Pang GL, Luo W, Tu JC, Zheng F, Liu SM, Han L, Zhang JK, Luo XY, Zhou X (2014). MiRNA-15a mediates cell cycle arrest and potentiates apoptosis in breast cancer cells by targeting synuclein-gamma. Asian Pac J Cancer Prev.

[25] Vance KW, San-som SN, Lee S, Chalei V, Kong L, Cooper SE, Oliver PL, Ponting CP (2014). The long non-coding RNA Paupar regulates the expression of both local and distal genes. The EMBO journal.

[26] Li Z, Chao TC, Chang KY, Lin N, Patil VS, Shimizu C, Head SR, Burns JC, Rana TM (2014). The long noncoding RNA THRIL regulates TNFalpha expression through its interaction with hnRNPL. Proc Natl Acad Sci USA.

[27] Wilusz CJ, Wilusz J (2012). HuR and translation–the missing linc(RNA). Mol Cell.

[28] Yoon JH, Abdelmohsen K, Srikantan S, Yang X, Martindale JL, De S, Huarte M, Zhan M, Becker KG, Gorospe M (2012). LincRNA-p21 suppresses target mRNA translation. Mol Cell.

[29] Huang JF, Guo YJ, Zhao CX, Yuan SX, Wang Y, Tang GN, Zhou WP, Sun SH (2013). Hepatitis B virus X protein (HBx)-related long noncoding RNA (lncRNA) down-regulated expression by HBx (Dreh) inhibits hepatocellular carcinoma metastasis by targeting the intermediate filament protein vimentin. Hepatology.

[30] Lan X, Yan J, Ren J, Zhong B, Li J, Li Y, Liu L, Yi J, Sun Q, Yang X, Sun J, Meng L, Zhu W (2016). A novel long noncoding RNA Lnc-HC binds hnRNPA2B1 to regulate expressions of Cyp7a1 and Abca1 in hepatocytic cholesterol metabolism. Hepatology.

[31] Cao C, Sun J, Zhang D, Guo X, Xie L, Li X, Wu D, Liu L (2015). The long intergenic noncoding RNA UFC1, a target of MicroRNA 34a, interacts with the mRNA stabilizing protein HuR to increase levels of beta-catenin in HCC cells. Gastroenterology.

[32] Yang F, Zhang L, Huo XS, Yuan JH, Xu D, Yuan SX, Zhu N, Zhou WP, Yang GS, Wang YZ, Shang JL, Gao CF, Zhang FR (2011). Long Noncoding RNA High Expression in Hepatocellular Carcinoma Facilitates Tumor Growth Through Enhancer of Zeste Homolog 2 in Humans. Hepatology.

[33] Du Z, Sun T, Hacisuleyman E, Fei T, Wang X, Brown M, Rinn JL, Lee MG, Chen Y, Kantoff PW, Liu XS (2016). Integrative analyses reveal a long noncoding RNA-mediated sponge regulatory network in prostate cancer. Nat Commun.

