# Supplementary material for: Long non-coding RNA AK058003, as a precursor of miR-15a, interacts with HuR to inhibit the expression of Υ-synuclein in hepatocellular carcinoma cells
Source: Oncotarget. 2016 Dec 27;8(6):9451–65. doi: 10.18632/oncotarget.14276 (PMC5354744; doi:10.18632/oncotarget.14276)
Supplement: Supplementary file 1 [file oncotarget-08-9451-s001.pdf]

Long non-coding RNA AK058003, as a precursor of miR-15a, interacts with HuR to inhibit the expression of γ-synuclein in hepatocellular carcinoma cells

Supplementary Materials

Supplementary Table S1: miRNA binding sites of SNCG by starBase v2.0

Position	Seq-target	Name
42	TGGCAGTGTCTTAGCTGGTTGT	hsa-miR-34a-5p
62	ACAGCAGGCACAGACAGGCAGT	hsa-miR-214-3p
62	AGCAGCATTGTACAGGGCTATCA	hsa-miR-107
42	AGGCAGTGTAGTTAGCTGATTGC	hsa-miR-34c-5p
62,77	TAGCAGCACGTAAATATTGGCG	hsa-miR-16-5p
62,77	TAGCAGCACATAATGGTTTGTG	hsa-miR-15a-5p
42	AGGTTGTCCGTGGTGAGTTCGCA	hsa-miR-323b-5p
62,77	TAGCAGCACAGAAATATTGGC	hsa-miR-195-5p
42,62,77	CAGCAGCACACTGTGGTTTGT	hsa-miR-497-5p
62	TTCACAGTGGCTAAGTTCCGC	hsa-miR-27a-3p
62	AGCAGCATTGTACAGGGCTATGA	hsa-miR-103a-3p
39	TAAGGCACGCGGTGAATGCC	hsa-miR-124-3p
62,77	TAGCAGCACATCATGGTTTACA	hsa-miR-15b-5p
42	TGGCAGTGTATTGTTAGCTGGT	hsa-miR-449a
42	AGGCAGTGTATTGTTAGCTGGC	hsa-miR-449b-5p
62	TTCACAGTGGCTAAGTTCTGC	hsa-miR-27b-3p
62,77	CAGCAGCAATTCATGTTTTGAA	hsa-miR-424-5p

Supplementary Table S2: The sequence of siRNA

Gene symble	Sequence
lncRNA-AK058003-si1	
sense	5′-CCACCAGUUACCUGCAAUATT dTdT-3′
antisense	3′-dTdT TTGGUGGUCAAUGGACGUUAU-5′
lncRNA-AK058003-si2	
sense	5′-GGAACAAAGAUGGUUUCUATT dTdT-3′
antisense	3′-dTdT TTCCUUGUUUCUACCAAAGAU-5′
SNCG-si1	
sense	5′-GGUGAGGCAUCCAAAGAGA dTdT-3′
antisense	3′-dTdT CCACUCCGUAGGUUUCUCU-5′
SNCG-si2	
sense	5′-GACCAAGGAGAAUGUUGUA dTdT -3′
antisense	3′-dTdT CUGGUUCCUCUUACAACAU-5′
SNCG-si3	
sense	5′- UGACGGAAGCAGCUGAGAA dTdT -3′
antisense	3′-dTdT ACUGCCUUCGUCGACUCUU-5′

Supplementary Table S3: Genes and primer sequences

Gene symbol	Sequence(from 5′ to 3′)
LncRNA-AK058003	F: GGGAACAAAGATGGTTTCTACGT
	R: ACTGGTTCATAGTTAGGCTGGAT
SNCG	F: CGTGAGCGAGGCTGTGGTGA
	R:TCCTCTGCCACTTCCTCTTTCT
HuR	F:TCCGGCTGGTGCATTTTCAT
	R:AGTCGCGGATCACTTTCACA
MiR-15a	F:TGCGCTAGCAGCACATAATGGT
	R:CCAGTGCAGGGTCCGAGGTATT
	Loop:GTCGTATCCAGTGCAGGGTCCGAGGTATTCGCACTGGATA CGACCACAAAC
GAPDH	F: AGAAGGCTGGGGCTCATTG
	R: AGGGGCCATCCACAGTCTTC
β-actin	F:AGCGAGCATCCCCCAAAGTT
	R:GGGCACGAAGGCTCATCATT
U6	F:CTCGCTTCGGCAGCACA
	R:AACGCTTCACGAATTTGCGT
LncRNA-AK058003 fragement	
1-1197nt	F:GGCATGGTGGCTCATGCCTGTACTC
421-1197nt	F:GGTTTCTACGTGCTTCCAGAG
776-1197nt	F:CTCAAGTGACTGAGGTGGG
1197-1176nt	R:GAGACGGAGTCTCGCCCTTTC
Aantisence	F:GAGACGGAGTCTCGCCCTTTC
	R:GGCATGGTGGCTCATGCCTGTAC

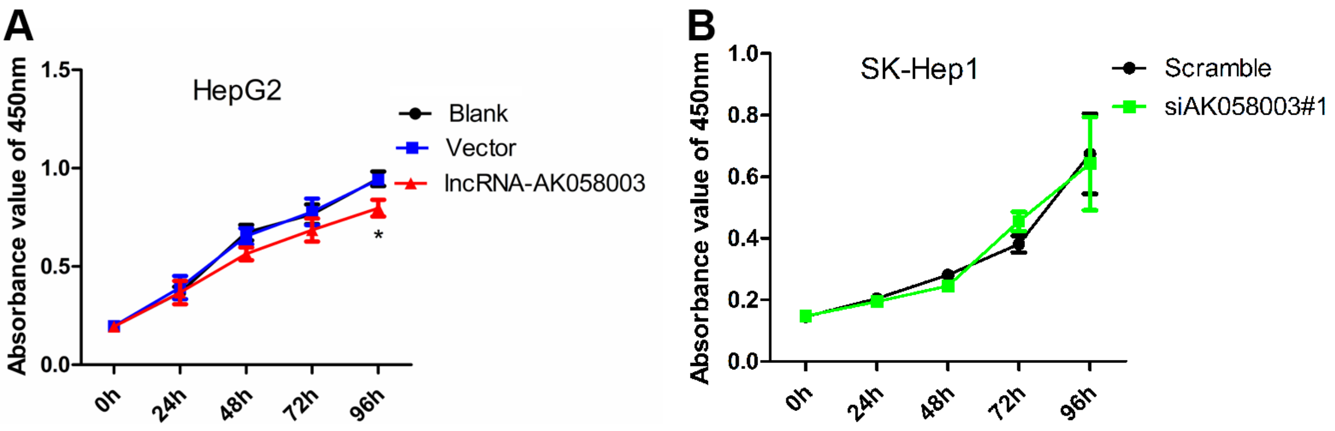

Supplementary Figure S1: (**A**) CCK8 assays showed that cell proliferation was suppressed by lncRNA-AK058003 overexpression in HepG2 cells. (**B**) The proliferation curves were not significantly different in lncRNA-AK058003 down-regulated SK-HeP1 cells. $*p < 0.05$.

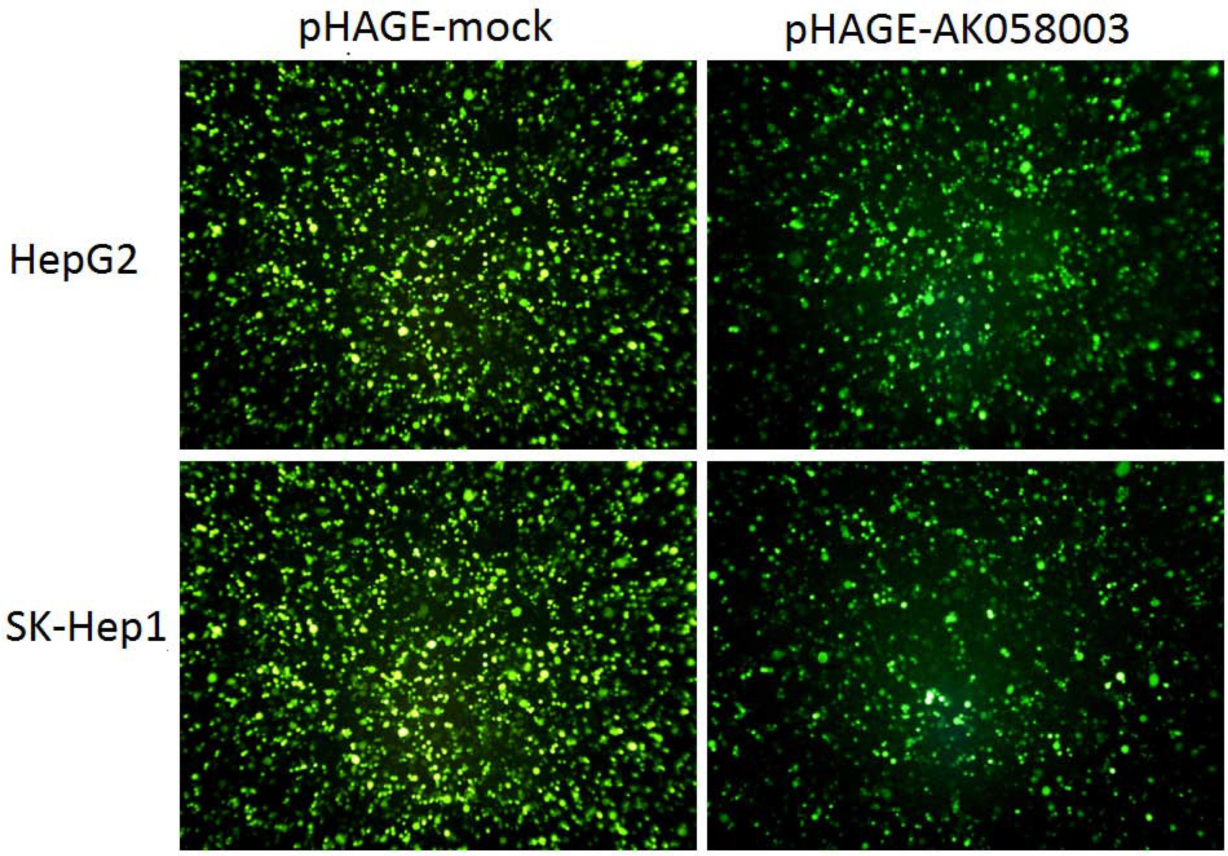

Supplementary Figure S2: The infection efficiency of the recombinant lentivirus (pHAGE) containing human lncRNA-AK058003 gene groups or pHAGE-mock groups in HepG2 and SK-HeP1 cells were determined by fluorescent microscopy. Original magnification, ×100. Data based on three independent experiments.

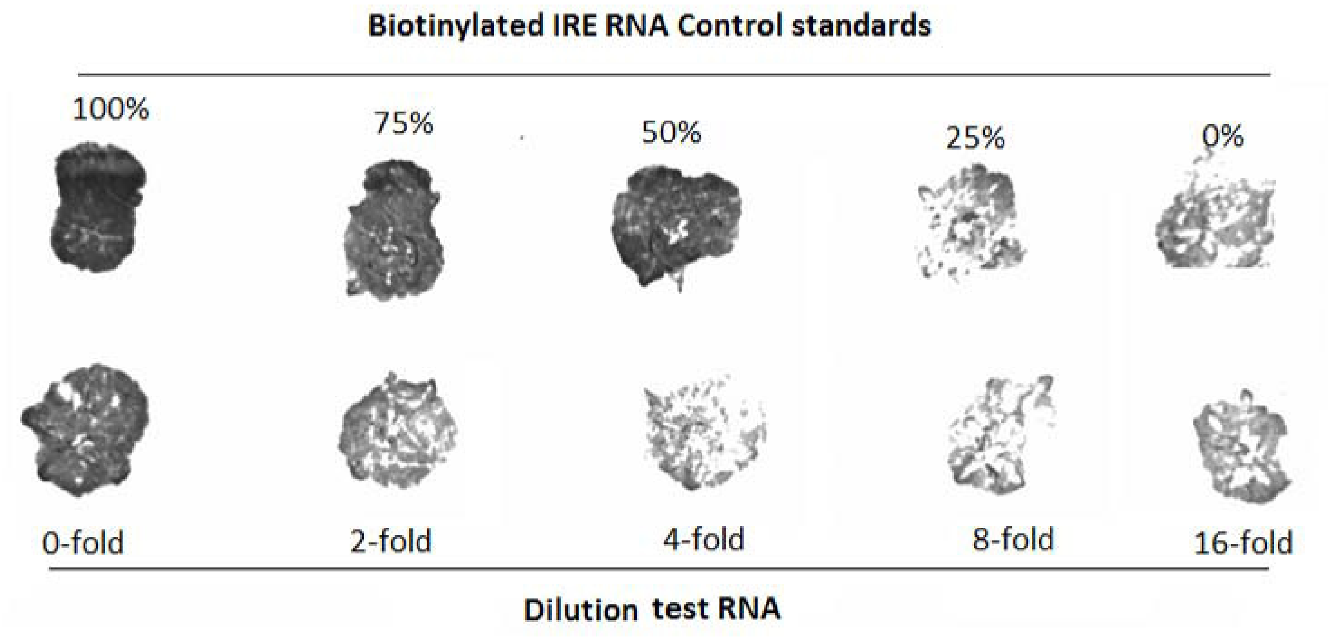

Supplementary Figure S3: Biotinylated RNA efficiency by dot blotting analysis is shown. A series of concentrations of biotinylated IRE RNA acted as standards.

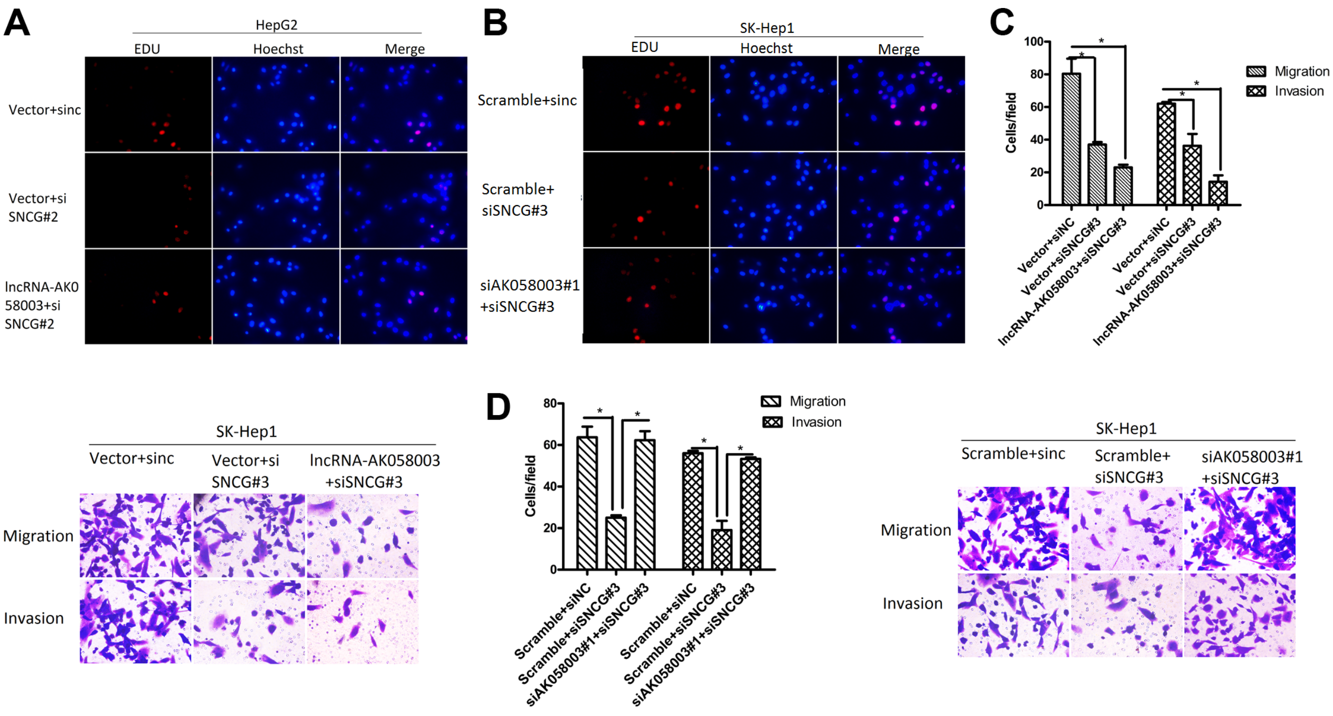

Supplementary Figure S4: (**A**) LncRNA-AK058003 and siSNCG co-transfection in HepG2 cells and (**B**) siAK058003#1 and siSNCG co-transfection in SK-HeP1 cell lines were seeded into 96 well plates and cell proliferation was assessed by EdU immunofluorescence staining. Original magnification, ×400. The barplot on the right shows the percentage of EdU positive nuclei. (**C**, **D**) Transwell migration and invasion assays of SK-Hep1 cells were determined after co-transfection of lncRNA-AK058003 + siSNCG or siAK058003 + siSNCG. The graph on the left shows the positive cells per field . Original magnification, ×400. Data are shown as the mean ± S.E.M. based on three independent experiments.*$p < 0.05$, **$p < 0.01$,***$p < 0.001$.

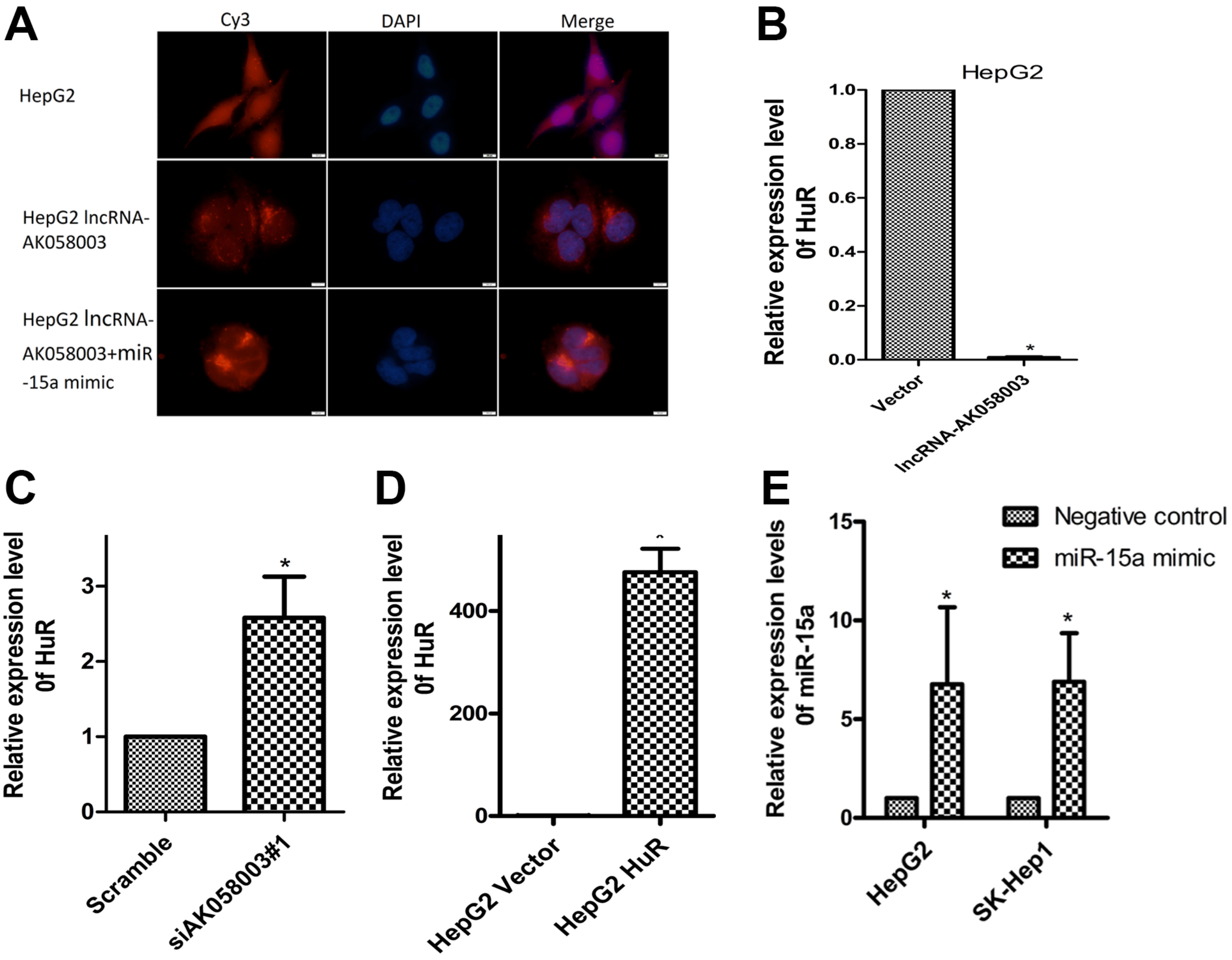

Supplementary Figure S5: (**A**) RNA FISH detection of lncRNA-AK058003. Fixed HepG2 cells with DAPI-stained nuclei (blue) were probed simultaneously for lncRNA-AK058003. Original magnification, ×1000. (**B**, **C**) The expression of HuR had a negative correlation with lncRNA-AK058003. (**D**) qRT-PCR analysis shows the overexpressed level of HuR. (**E**) The expression of miR-15a in HCC cells after transfected miR-15a mimics. Data are shown as the mean ± S.E.M. based on three independent experiments.*$p < 0.05$, **$p < 0.01$,***$p < 0.001$.
